# Participation in Tobacco Cessation Programs Among Medicaid Managed Care Enrollees in Florida

**DOI:** 10.3390/healthcare12222319

**Published:** 2024-11-20

**Authors:** Rahma S. Mkuu, Casey C. Glymph, Peyton A. Lurk, Madison R. McCraney, Jennifer H. LeLaurin, Ramzi G. Salloum, Jaclyn M. Hall, Christopher R. Cogle

**Affiliations:** 1 Department of Health Outcomes and Biomedical Informatics, College of Medicine, University of Florida, Gainesville, FL 32610, USA; rmkuu@ufl.edu (R.S.M.);; 2 Department of Medicine, College of Medicine, University of Florida, Gainesville, FL 32610, USA; 3 College of Social Sciences and Public Policy, Florida State University, Tallahassee, FL 32306, USA

**Keywords:** tobacco cessation, Medicaid, managed care

## Abstract

**Background/Objectives**: Tobacco use remains a significant public health issue, particularly among individuals with low incomes, including Medicaid recipients who often face multiple barriers to quitting. This study aimed to identify barriers, from the perspective of Medicaid managed care organizations (MCOs), influencing Medicaid recipient participation in tobacco cessation programs. **Methods**: Focus group interviews were conducted with Florida Medicaid MCOs to elicit processes for case identification, outreach, referral, program participation, and incentives. Answers were synthesized into themes. **Results**: Medicaid recipients were primarily identified through nicotine dependency claim codes or Health Risk Assessments (HRAs). Individuals were referred to state and local community tobacco cessation programs through text messaging and outreach by MCO case managers. The MCOs identified the following as barriers: primary care physicians (PCPs) with limited knowledge about cessation programs and pharmacologic treatments for nicotine dependence, low availability of health coaches, long wait times for entry into cessation programs, weak coordination between MCOs and cessation programs, and insufficient incentives for individuals for program participation. Suggested strategies to overcome barriers were continuing medical education (CME) for PCPs about tobacco cessation programs and prescription therapies, increasing the training of health coaches, more investment in quitlines, increasing data sharing between MCOs and cessation programs, and increasing incentives for individuals. **Conclusions**: These findings highlight the importance of engaging MCOs in discussions about policy and program improvements, as their insights can drive meaningful changes in how tobacco cessation and other preventive health programs are structured and implemented. Targeted interventions are needed to enhance tobacco cessation program participation among Medicaid recipients.

## 1. Introduction

Tobacco use remains a critical public health issue and leads to more preventable deaths in the United States (U.S.) each year than HIV, illegal drug use, alcohol use, motor vehicle injuries, and firearm-related incidents combined [1]. Tobacco initiates and exacerbates numerous chronic diseases such as cancer, asthma, heart disease, and stroke [1]. The harmful effects of smoking have the largest impact on vulnerable populations, especially those of low socioeconomic status who may have health insurance coverage by a state Medicaid program [1,2]. Despite a gradual decrease in smoking in the overall U.S. population, smoking rates of Medicaid recipients are twice the rate of adults with private health insurance [3,4].

Recognizing the potential to prevent costly diseases, the Florida Legislature in 2011 mandated that Medicaid MCOs establish a suite of lifestyle improvement programs called Healthy Behaviors for individuals enrolled in Medicaid health plans (§409.973(3), Florida Statute) [5]. Per statute, the Medicaid MCOs must provide access to a “medically approved smoking cessation program,” which in this report is termed “tobacco cessation program” to comport with contemporary understandings and efforts. In 2014, Florida Medicaid MCOs began coverage of tobacco cessation services and supports, which were voluntary programs requiring written consent to participate. How the MCOs operationalized access to tobacco cessation has yet to be described.

Two years after the tobacco cessation program implementation, among 4,561,975 Medicaid recipients, only 338 people were enrolled in a tobacco cessation program and only 62 completed a program [2]. While the underlying reasons for such low participation remain unclear, it is essential to recognize that the managed care organizations (MCOs) are responsible for administering these programs and identifying eligible individuals. Despite their initial efforts, the results indicate significant challenges in translating these efforts into meaningful program participation.

This study aimed to define Florida’s Healthy Behaviors tobacco cessation implementation, determine barriers to participation from the perspective of Florida Medicaid MCOs, and identify strategies to address those barriers. Overcoming barriers to Medicaid recipients’ participation in tobacco cessation programs is crucial for reducing the disproportionate burden of tobacco-related diseases among vulnerable populations, improving public health outcomes, and ultimately lowering healthcare costs associated with preventable chronic conditions.

## 2. Methods

This study was reviewed by the University of Florida Institutional Review Board and was determined to qualify as non-human exempt research under federal regulations (protocol number NH00043177). A qualitative study design was used to determine how Medicaid MCOs in Florida identified and engaged individuals for their tobacco cessation programs [3,4]. All Florida Medicaid MCOs with health plans were invited by email to discuss how they implemented the statute-mandated Healthy Behaviors tobacco cessation program. In separate one-hour videoconferences with each MCO in April 2023, we interviewed a minimum of two staff members, including at least one physician, who were directly responsible for tobacco cessation services. The interviews collected information about how the MCO identified and referred individuals for tobacco cessation programs, types of tobacco cessation programs used (i.e., local community programs, state-supported program, 1-800-QUIT-NOW, distinct MCO services and supports), partners they collaborated with, and barriers to increasing individual participation from the perspective of the MCO. Representatives from the MCOs held various positions in care management, population health, and quality. A semi-structured interview guide was used, including open-ended questions (Supplementary Table S1). The guide allowed for flexibility in the discussion, enabling MCO staff to elaborate on their experiences and insights.

Interview notes were de-identified to ensure the anonymity of MCOs. Responses were coded by two independent coders into themes [6]. The coders first independently coded the notes, after which they met to discuss their findings. Any discrepancies between the coders were resolved through discussion, and, when necessary, a third coder was consulted to reach a consensus. Coding was conducted iteratively, with themes refined and adjusted as needed throughout the process to ensure accuracy and depth in the analysis.

## 3. Results

Six of the eight Florida Medicaid MCO health plans participated in interviews. The six MCOs managed the health care of 75% of Medicaid enrollees in the state. Two of the six MCOs covered Medicaid enrollees throughout the state, while four of six provided regional coverage. All eleven regions of the state were represented in this study by a minimum of three MCOs per region. The MCOs in this report varied in terms of company size and number of Medicaid enrollees, providing a breadth of perspective on Medicaid service delivery in Florida.

In common, all six MCOs verified coverage for tobacco cessation services in compliance with state statute. However, there was great variation in how the MCOs provided access and supported the utilization of tobacco cessation programs (Table 1).

### 3.1. Programs and Partners

Most commonly, MCOs identified nicotine-dependent individuals through claim codes or annual Health Risk Assessments (HRAs). MCO case managers referred these identified individuals to the state-supported tobacco cessation program called Tobacco Free Florida, the U.S. Department of Health and Human Services (HHS) supported 1-800-QUIT-NOW program, and/or local area health education centers (AHECs), which offered group therapy sessions.

Most MCOs used health coaches to provide periodic support through phone calls to nicotine-dependent individuals. Health coaches were described as tobacco treatment specialized clinicians, such as registered nurses (RNs) or mental health therapists (i.e., licensed clinical social workers and licensed mental health counselors). The MCOs reported that the number of individuals participating in tobacco cessation programs was dependent on the number of employed health coaches and that there was limited availability of health coaches, which limited the number of program participants. From the MCO experience, optimal tobacco cessation involved a combination of health coaching, educational sessions from the state or community partner, and support from primary care physicians (PCPs).

Although Nicotine Replacement Therapy (NRT) was covered by all MCOs, only two of six proactively dispensed the NRTs to the homes of individuals. Pharmacological options were covered and dependent on shared decision-making between the individual patient and physician.

### 3.2. Incentives

Incentives were previously capped at USD 50 per recipient per year for all Healthy Behavior programs by the state Medicaid agency. The MCOs distributed incentives at various stages of program participation, such as initiation, mid-program, and completion. These incentives primarily came in the form of gift cards to drug stores, online stores, and merchandise reward redemption stores. There were minor differences in dollar amounts provided (Table 1). MCOs reported that their enrollees perceived the value of these incentives as low.

### 3.3. Barriers and Strategies

The MCOs identified several barriers to the Healthy Behaviors program’s success (Table 2). The MCOs reported that primary care physicians (PCPs) lacked adequate knowledge about tobacco cessation programs, pharmacologic options to support tobacco cessation, and patient-centered resources for quitting tobacco. Additionally, the 1-800-QUIT-NOW program had a wait time of up to three weeks, which discouraged individuals from using this resource. The MCOs reported that the emerging issue of children using electronic nicotine devices (ENDs) necessitated targeted interventions for their enrollees and that there were limited resources for educating children and adolescents about the dangers of e-cigarette or vaping use-associated lung injury (EVALI), which has been linked to vitamin E acetate, a synthetic form of vitamin E often found in illicit (tetrahydrocannabinol) THC vaping products. Furthermore, there appeared to be poor coordination between partnering tobacco cessation programs and MCOs. The lack of coordination impaired the tracking of Medicaid recipient progress, cessation outcomes, healthcare utilization, and health outcomes.

## 4. Discussion

This is the first report describing MCO perspectives on implementing statute-mandated tobacco cessation programs for Medicaid recipients. While the MCOs were responsible for providing access to tobacco cessation programs, they reported that systemic barriers, such as insufficient knowledge among primary care physicians (PCPs), challenge in hiring health coaches, and difficulties with navigating program websites, indirectly affected their ability to facilitate Medicaid enrollee participation. Understanding the viewpoint of MCOs is crucial for several reasons. First, MCOs in the United States serve a pivotal role in shaping the delivery and accessibility of healthcare services, including tobacco cessation programs. By capturing their perspectives, our study sheds light on practical barriers and operational challenges that are often overlooked in traditional research focused solely on recipients or providers. This approach provides a comprehensive understanding of the systemic factors that influence program participation and effectiveness. While past reports plainly criticized Medicaid MCOs for not implementing comprehensive tobacco cessation [7], this study sought to understand the pragmatic barriers and potential solutions from the policy implementers—the MCOs. Our findings indicate that low participation in tobacco cessation programs is due to several factors, including limited clinician knowledge about tobacco cessation, long waiting periods that restrict access, lack of standardization in Healthy Behaviors across MCOs, poor integration between MCOs and state and community programs, and inadequate incentives for Medicaid recipients to enroll in or complete cessation programs.

The MCOs used a wide variety of methods to implement statute-mandated Healthy Behaviors access to tobacco cessation programs. An alternative to this heterogeneous implementation could be a standardized approach across all MCOs. By standardizing the Healthy Behaviors access to tobacco cessation programs there may come the benefits of consistency in quality programming, improved program accessibility, streamlined training for physicians, enhanced data collection and analytics, cost efficiency, stronger partnerships with organizations like the state’s Tobacco Free Florida (TFF) and local AHECs, and simplified marketing.

The MCOs reported that a major limitation was the insufficient number of health coaches—clinicians with certification or specialization in healthy lifestyle promotion and disease prevention—available to recruit, promote, and sustain engagement in community programs. In addition, this limitation has been reported from a program perspective as a barrier to providing tobacco cessation services for Medicaid recipients [8]. The availability of health coaches is a significant workforce deficiency because of evidence showing that proactive tobacco treatment intervention is associated with people more likely to use cessation medications and more likely to successfully quit tobacco compared to usual care [9]. MCOs could sponsor training of RNs, LCSWs, and LMHCs to become certified tobacco treatment specialists (TTSs), which would increase the availability of health coaches. Additionally, state workforce support efforts often focus on resident physicians and medical students, whereas adding health coaches to workforce support efforts should be considered given their important role in preventing disease and the need for physicians.

At the clinical level, some physicians were perceived to have limited knowledge about MCO tobacco cessation programs and benefits. This is significant because a physician referring a person to a tobacco cessation program is associated with an increased likelihood that the person participates in the cessation program [10,11]. In a prior study, the most common barrier for physicians providing tobacco cessation resources or treatment was a lack of knowledge, followed by a need for more training on tobacco cessation programs [12]. In one survey, only half of physicians addressed tobacco directly with their patients, advised patients to quit at every visit, or provided tobacco cessation advice [13]. Less than 10% arranged follow-ups to discuss tobacco cessation progress. Providing healthcare professionals with a script to use while assessing interest in quitting, advising, and providing resources to patients could be helpful in increasing participation in tobacco cessation programs. Another solution is to employ clinic-embedded or online-ready health coaches to counsel patients immediately after their doctor visits.

At the patient level, MCOs reported that low value incentives likely dampened enthusiasm to participate. Quitting tobacco provides the individual an internal incentive for better health, yet external incentives in the form of payment increase engagement and prolong smoking cessation [14]. From the MCO perspective, Florida’s current cap of USD 50 per person per year was deemed too low a financial incentive to participate in tobacco cessation. Moreover, USD 50 is less than the incentive amount suggested by studies showing the effectiveness of financial incentives for tobacco cessation [7,15,16]. Financial incentives are particularly effective motivators for tobacco cessation among Medicaid recipients [17]. Furthermore, the MCOs highlighted that their Medicaid enrollees often had more than one preventable lifestyle risk, like tobacco, obesity, and substance use. Prior reports show that discussing one preventive service increases a patient’s interest in other preventive services [16]. Therefore, increasing and separating financial caps on incentives was suggested by the MCOs to increase participation in tobacco cessation and other Healthy Behavior programs.

In our study, the completion rates of tobacco cessation programs among Medicaid recipients were low, a finding consistent with broader trends reported in the literature. For instance, a large prospective national survey found that less than 1% of patients utilized tobacco cessation programs following exacerbations of chronic obstructive pulmonary disease (COPD), despite these individuals being among the highest beneficiaries of such interventions [18]. Similarly, research has shown that cancer patients also experience low participation rates in tobacco cessation programs, with studies indicating that only 7% of these patients engage in available cessation services, despite the clear benefits of quitting for their treatment outcomes [19]. These comparisons underscore the substantial gap in engagement with cessation programs across populations at high risk for tobacco-related morbidity and mortality. They suggest that the barriers to participation are systemic and pervasive, necessitating more robust efforts to enhance access, awareness, and motivation for patients to engage in these critical health interventions.

When interpreting the findings of this study, it is important to consider certain limitations. First, reported barriers from MCO representatives may not provide a comprehensive representation of the barriers from the individual Medicaid recipient perspective. Second, the study’s findings are primarily based on the experiences of MCOs in Florida, which limit the generalizability of the results to other states with different Medicaid policies. Another limitation was the paucity of information from the MCOs about ENDs as a distinct health behavior requiring unique prevention strategies. Another potential weakness is the use of self-reported data from MCO staff, which may be subject to recall bias or incomplete information. Furthermore, while the study identifies key barriers, it does not provide direct evidence of the effectiveness of proposed solutions, such as increasing incentives or expanding health coaching, making further investigation necessary to validate these strategies. While our current study focused on barriers to participation in Medicaid-sponsored tobacco cessation programs, we recognize the importance of exploring other strategies tobacco users may be using to quit, such as self-directed approaches, informal support networks, or alternative nicotine products. Incorporating these other strategies could enhance the relevance and flexibility of cessation programs. Another limitation of this study is that we were unable to access data on the number of referrals made to tobacco cessation programs from the MCOs, which restricts our ability to assess the full scope of the referral process. Instead, we only had access to data on the number of Medicaid recipients who were enrolled in and completed the programs, leaving a gap in understanding how many potential participants may have been referred but did not engage. Despite these limitations, the barriers and strategic recommendations provide valuable information to change Medicaid policy, plan management, and quality oversight of statute-mandated programs like Healthy Behaviors.

## 5. Conclusions

Addressing the barriers identified in this study is important for improving participation in tobacco cessation programs among Medicaid recipients, a population disproportionately affected by tobacco-related health issues. The findings underscore the need for standardized approaches across Medicaid MCOs to ensure consistent quality and accessibility of cessation programs. Enhancing physician education on tobacco cessation resources, increasing the use of health coaches, strengthening coordination between MCOs and tobacco cessation programs, and offering higher-value incentives are key strategies that could significantly boost enrollment and completion rates. By implementing these targeted strategies, Medicaid programs can better support individuals in quitting tobacco, ultimately leading to improved health outcomes and reduced healthcare costs for this vulnerable population. These findings also highlight the importance of engaging MCOs in discussions about policy and program improvements, as their insights can assist in how tobacco cessation and other preventive health programs are structured and implemented.

## Figures and Tables

**Table 1:** Summary of Managed Care Organization (MCO) access to tobacco cessation programs.

MCO Number	Program Description	Methods of Case Identification and Contact	Incentives
1	Health coaches who were RNs specialized in tobacco cessation. Followed the 5 As (ask, advise, assess, assist, arrange follow-up) model of tobacco treatment.	Claim data, healthy behavior mailers, outreach through telephone and mail, remote patient monitoring, transportation vendors, medical case management, provided tele-health behavioral health services, software system to track patient progress.	Cessation program enrollment: merchandise worth USD 5–15; mid-program: merchandise worth USD 5–15; completion: merchandise worth USD 5–20.
2	Health coaches and care managers including registered nurses (RNs), licensed mental health counselors (LMHCs), and licensed clinical social workers (LCSWs). Focused on achievable goals, sustaining motivation, and monitoring progress.	Claim data, substance use dashboards, health risk assessments, care management program.	Enrollment: USD 25 drugstore gift card; completion: additional USD 25 gift card.
3	Partnered with local Area Health Education Centers (AHECs) for group therapy. Partnered with state cessation services through Tobacco Free Florida (TFF) for quitline, online, and group therapy. Participants received printed strategies to quit and free over-the-counter Nicotine Replacement Therapy (NRT). Health coach system for managing calls for various health programs. Case management for smokers.	Community outreach events, AHEC referrals, TFF referrals, Health Risk Assessments (HRA) of individuals.	Completion: USD 50 gift card.
4	MCO provided eight health coaching sessions via phone. Mailed a three-month supply of over-the-counter NRT to individuals’ homes.	Health risk assessments, monthly text messaging to identify members.	After two sessions: USD 25 gift card; after completing all eight sessions: another USD 25 gift card.
5	Care coordinators or managers referred individuals to quit smoking through referral to AHEC for single and multi-session group therapy.	Claim data, patient management software system, risk score, text messaging.	Health Risk Assessment completion: USD 10 reward; initial session completion: USD 10; six sessions completion: USD 30 reward.
6	Individuals chose their own tobacco cessation program using their own research or recommendations from the MCO. Individuals submitted a certificate of completion documentation of attendance and completion.	Self-referrals, physician referrals, claim data, and medical record reviews. Included a dedicated health behavior specialist. Vendor provided chip rewards and over-the-counter NRT. Designed for adults 18+ with web-based support and text messaging.	Attendance certificate: USD 50 gift card; tobacco-free for one month: USD 20 gift card; three months: additional USD 20 gift card with PCP attestation.

**Table 2:** Barriers and strategies as perceived by MCOs for increasing Medicaid recipient participation in tobacco cessation programs.

Barriers from MCO Perspective	Strategies by MCOs
PCPs with low knowledge of tobacco cessation programs and pharmacologic options for tobacco cessation.	Provide free continuing medical education (CME) for PCPs about tobacco cessation and pharmacologic options.
Prolonged wait time for enrolling in 1-800-QUIT-NOW.	Increased investments by U.S. Department of Health and Human Services and states into 1-800-QUIT-NOW.
Low value of incentives for individuals to initiate or complete tobacco cessation programs.	Increase incentive caps by state Medicaid agency and CMS. Allow each Healthy Behavior program to have its own cap and ability to combine incentives so that individuals with multiple needs for lifestyle changes can be incentivized.
Challenges in engaging individuals with mental illness and tobacco use.	Colocation of mental health and tobacco cessation services. Free continuing education units (CEU) for mental health therapist education about tobacco cessation programs. Engage peer support groups and peer navigators with lived experience.
Poor connection with community tobacco cessation programs.	Establish secure data sharing between MCOs and state and local tobacco cessation programs to improve patient referrals and communication about patient progress, completion, and outcomes.
Challenges for individuals with low socioeconomic resources to adhere to program requirements.	Shorten program duration. Increase flexibility of scheduling. Increase flexibility of participation.
Rising prevalence of ENDs.	Intensify efforts in ENDs prevention among children. Outreach to schools.

## Data Availability

The original contributions presented in the study are included in the article, further inquiries can be directed to the corresponding author.

## Supplementary Materials

The following supporting information can be downloaded at: https://www.mdpi.com/article/10.3390/healthcare12222319/s1, Table S1. Open-Ended Questions Used in Interviews with Managed Care Organizations about Access and Utilization of Tobacco Cessation Programs.
