# Supplementary material for: Participation in Tobacco Cessation Programs Among Medicaid Managed Care Enrollees in Florida
Source: Healthcare (Basel). 2024 Nov 20;12(22):2319. doi: 10.3390/healthcare12222319 (PMC11593654; doi:10.3390/healthcare12222319)
Supplement: Supplementary file 1 [file healthcare-12-02319-s001.zip › healthcare-3233424-supplementary.pdf]

Supplementary Table S1. Open-Ended Questions Used in Interviews with Managed Care Organizations about Access and Utilization of Tobacco Cessation Programs.

1. **How are potential individuals identified?** Methodological approaches and criteria are utilized by each MCO to identify individuals for tobacco cessation program participation.
2. **How is the program implemented?** Details on implementation strategies employed by the MCOs for their tobacco cessation programs. MCOs were asked to share their referral process, their team involved in referring and supporting individuals who participate, and the overall structure of the cessation program.
3. **How are members incentivized to participate?** This question focused on the types and value of incentives offered and how incentives were distributed by MCOs to their Medicaid recipients who enroll in tobacco cessation programs. Discussions were focused on milestones for receipt of incentives, the range of rewards, and how these incentives were distributed throughout the program.
4. **What is the role of community partners in increasing participation?** This question investigated the extent to which the MCOs collaborated with community partners, such as Tobacco-Free Florida (TFF) and Area Health Education Centers (AHECs). The discussion explored the nature of the collaborations, the services provided by the community partners, and the overall impact of the partnerships in engaging Medicaid recipients to participate and complete the programs.
5. **What are the current barriers to implementation?** This question solicited barriers that prevent individuals from participating in tobacco cessation programs. MCOs were encouraged to reflect on identification, referral, program expectations and requirements, incentives, and integration with healthcare.
6. **What are potential solutions to the barriers identified?** This question solicited strategies for overcoming barriers. The identification of barriers that preclude members from participating in tobacco cessation programs. MCOs were encouraged to reflect on barriers identified in the previous question.
